# Application of Advanced Molecular Methods to Study Early-Onset Neonatal Sepsis

**DOI:** 10.3390/ijms25042258

**Published:** 2024-02-13

**Authors:** Chrysoula Kosmeri, Vasileios Giapros, Anastasios Serbis, Maria Baltogianni

**Affiliations:** 1 Department of Pediatrics, University Hospital of Ioannina, 45500 Ioannina, Greece; 2 Neonatal Intensive Care Unit, School of Medicine, University of Ioannina, 45500 Ioannina, Greece

**Keywords:** early-onset sepsis, molecular methods, 16S rRNA, microRNA, bioinformatics

## Abstract

Early-onset sepsis (EOS) is a global health issue, considered one of the primary causes of neonatal mortality. Diagnosis of EOS is challenging because its clinical signs are nonspecific, and blood culture, which is the current gold-standard diagnostic tool, has low sensitivity. Commonly used biomarkers for sepsis diagnosis, including C-reactive protein, procalcitonin, and interleukin-6, lack specificity for infection. Due to the disadvantages of blood culture and other common biomarkers, ongoing efforts are directed towards identifying innovative molecular approaches to diagnose neonates at risk of sepsis. This review aims to gather knowledge and recent research on these emerging molecular methods. PCR-based techniques and unrestricted techniques based on 16S rRNA sequencing and 16S–23S rRNA gene interspace region sequencing offer several advantages. Despite their potential, these approaches are not able to replace blood cultures due to several limitations; however, they may prove valuable as complementary tests in neonatal sepsis diagnosis. Several microRNAs have been evaluated and have been proposed as diagnostic biomarkers in EOS. T2 magnetic resonance and bioinformatic analysis have proposed potential biomarkers of neonatal sepsis, though further studies are essential to validate these findings.

## 1. Introduction

Neonatal sepsis is a clinical syndrome characterized by signs or symptoms of infection within the first 28 days of life. This condition significantly contributes to morbidity and mortality among neonates, particularly those with very low birth weight and preterm infants [1,2,3]. A recent systematic review and meta-analysis of population-based studies from around the world found a population-level estimate for neonatal sepsis of 3900 cases per 100,000 livebirths and a mortality rate of 17.6%, ranging from 10.3 to 28.6% worldwide [4]. This study defined neonatal sepsis as cases that fullfilled International Consensus Conference on Paediatric Sepsis Definitions or modified clinical criteria [4].

Neonatal sepsis is categorized as early-onset sepsis (EOS) when it occurs within the initial 72 h of birth and as late-onset (LOS) sepsis when symptoms manifest at or after 72 h until 28 days of life. This review will focus on EOS, which remains a prominent global health concern due to its status as a leading cause of neonatal mortality [5,6]. It is mainly caused by Streptococcus agalactiae (group B streptococci, GBS) and Escherichia coli. Documented incidence of culture-proven EOS ranges between 0.77 and 1 per 1000 deliveries, with mortality rates reaching 20% [7,8,9]. The rate of EOS is increased among preterm infants, with rates of 3.5% for those born before 25 weeks of gestational age, 1.5% for those between the 25th and 28th weeks, and 1% for neonates born at 29 weeks or later [10,11,12]. The World Health Organization reports that 10% of annual deaths in children under 5 years old are attributed to neonatal sepsis [13].

Prompt diagnosis of EOS is of vital importance for early treatment and a better outcome. Diagnosing EOS remains challenging since the clinical signs and symptoms resemble those commonly observed in neonates during their transition from intrauterine to extrauterine life. EOS can happen in both preterm and term neonates. Despite efforts to use various clinical scores and expert opinions for EOS diagnosis, their diagnostic accuracy has been observed to be notably low in identifying neonatal sepsis [14]. A sepsis calculator, however, limited the use of antibiotic therapy for suspected EOS without missing cases of EOS, as shown in a meta-analysis of 13 studies analyzing a total of 175,752 newborns [15].

The gold standard for sepsis diagnosis is blood culture or other sterile body fluid culture accompanied by sensitivity tests of the pathogen to antibiotics [16]. Nevertheless, blood culture encounters several challenges in neonates, leading to its low sensitivity [17]. Firstly, drawing a sufficient amount of blood for culture proves difficult in neonates, particularly preterm infants. This special category that comprises around 10% of the total birth rate is vulnerable to infection because of an immature immune system. It has been found that 1 mL of blood is necessary for adequate sensitivity [18]. Furthermore, although exposing signs of infection, neonates may have low levels of bacteremia, while culturing requires 2 to 5 days to yield positive result. Blood culture may not yield a positive result due to infection with viruses, fungi, or anaerobes [19]. The last are not routinely checked in blood cultures during the neonatal period, and there are no commercially available anaerobic blood culture bottles validated for blood volumes under 3 mL [20]. The organisms not easily cultured under available laboratory conditions are called “microbial dark matter” in the literature [21].

The use of antibiotics by mothers before delivery also contributes to reduced sensitivity in blood culture [18]. Pregnant women at risk of premature delivery often receive multiple antibiotics, especially in cases of premature rupture of membranes, a common condition leading to preterm birth. The delay in pathogen identification results in broad-spectrum antibiotic usage, antibiotic resistance, secondary infections, and disturbed intestinal flora [22,23]. Changes in the evolving gut microbiome of infants are associated with the potential future risks of asthma, atopic conditions, obesity, and psychosocial diseases [24].

Other biomarkers that have been used in the diagnosis of sepsis are c-reactive protein (CRP), procalcitonin (PCT), and interleukin-6 (IL-6). However, most of these biomarkers are associated generally with inflammation and are not specific for infection [25]. These biomarkers are used as supplements to blood culture, and the use of a combination of several biomarkers has been recommended in order to achieve higher specificity and sensitivity [26].

Due to the disadvantages of blood culture and other common biomarkers, ongoing efforts are focused on exploring innovative molecular methods for the diagnosis and identification of neonates at risk of sepsis. These non-culture-based technologies include quantitative Polymerase Chain Reaction (PCR), 16S amplicon sequencing, and 16S–23S rRNA gene interspace region sequencing. The assessment of these novel molecular methods involves evaluating their sensitivity, specificity, and diagnostic accuracy to enable the early detection of sepsis in neonates. Molecular techniques, while presenting certain advantages, also have several drawbacks when compared to the conventional method of blood culture. Emerging techniques such as T2 magnetic resonance are also under evaluation for their applicability in neonatal sepsis. The aim of this review is to gather knowledge and recent research on these novel molecular methods in diagnosing sepsis and, specifically, EOS in neonates, which poses a great challenge to the clinician.

Furthermore, microRNA (miRNA) and bioinformatic analysis, by providing information about the host’s gene expression patterns, can identify infectious or inflammatory states. Therefore, although not able to detect microorganisms directly, they have been studied for their diagnostic accuracy and potential utility as diagnostic markers for EOS. This review will also present the available data regarding the use of miRNAs and bioinformatics in EOS.

## 2. Materials and Methods

The PubMed and Google Scholar databases were searched by using the following keywords: neonatal sepsis, EOS, molecular methods, molecular testing, diagnosis, diagnostic biomarkers, PCR, 16S rRNA, 16S-23S rRNA, microRNA, bioinformatics. We included peer-reviewed studies that were published until January 2024. The reference lists of the retrieved articles were also reviewed in search of other relevant articles that could have been missed in the initial search. Systematic reviews, meta-analyses, and observational studies were included if they referred to neonates.

## 3. PCR Techniques

Molecular diagnostic techniques, particularly PCR and various nucleic acid amplification methods, offer potential advantages in both diagnosing and treating neonatal sepsis [27]. These molecular methodologies primarily rely on PCR amplification techniques that target bacterial 16S or 23S rRNA genes and the 18S rRNA gene of fungi. The potential advantages and disadvantages of these novel molecular methods in contrast to traditional blood culture are discussed in Table 1.

In a systematic review, conducted in 2017, the evaluation of molecular testing utilized in neonatal sepsis encompassed a range of techniques, including broad-range PCR, real-time PCR, post-PCR processing, multiplex PCR, staphylococcal PCR, and fungal PCR [28]. The findings revealed a mean sensitivity of 0.90 and specificity of 0.93 for molecular tests in diagnosing neonatal sepsis, with evidence considered of moderate quality [28]. Notably, the mean estimated sensitivity of molecular assays surpassed that of other biomarkers, such as platelet count, CRP, PCT, tumor necrosis factor (TNF), and IL-6, while the mean specificity was comparable to these traditional tests [28]. The systematic review concluded that the currently available molecular assays might not possess sufficient diagnostic accuracy to replace microbial cultures. It is worth mentioning that the performance of these tests was compared to the reference standard, which is blood culture. However, considering the various drawbacks associated with blood culture, such as instances of culture-negative sepsis, a more meaningful comparison would be to assess molecular tests against clinical sepsis syndromes rather than relying solely on blood culture. This is particularly relevant because newborns can have sepsis that goes undetected through conventional culture methods. In addressing this challenge, molecular methods could potentially provide a more appropriate solution than traditional culture methods.

Furthermore, the absence of a universally applicable sepsis definition for the neonatal age group, adaptable to diverse resource settings and preterm cases, results in considerable differences among existing definitions of neonatal sepsis [9]. This variation poses a challenge to the comparability of studies on sepsis included in systematic reviews.

### 3.1. Quantitative or Real-Time PCR

A category of methods relies on targeted PCR to amplify and identify the DNA or RNA of specific organisms from bodily fluids, such as blood or cerebrospinal fluid [28]. However, these PCR-based assays exhibit certain limitations. Most importantly, they can detect a restricted number of microbial targets predetermined by the chosen PCR panel, rendering the identification of other pathogens unfeasible [21]. Additionally, considering the multitude of known antimicrobial resistance genes, quantitative PCRs can only identify resistance genes specifically targeted in the assay. Therefore, any novel mutations responsible for antimicrobial resistance would remain undetected [21]. Other drawbacks of this technique include high costs, delays in reporting up to one or more days, lack of standardization, and a complex procedure [21].

### 3.2. 16S rRNA PCR Testing

Innovative molecular techniques primarily utilize PCR amplification methods targeting bacterial 16S or 23S rRNA genes and the 18S rRNA gene of fungi. The widely shared 16S ribosomal gene, common to all bacteria [29], has been recognized as a defining factor for categorizing organisms as bacteria [30]. The 16S rRNA gene is 1500 nucleotides long, encodes the 30S ribosomal subunit in all prokaryotes, and demonstrates high conservation and stability over time. Utilizing gene-specific signatures, the 16S rRNA gene can accurately pinpoint specific bacteria [31,32], offering a cost-effective and rapid alternative to culture techniques [33].

In El-Amir’s study, involving 75 clinically suspected cases of neonatal sepsis, EOS was identified in 42.7% of cases, predominantly among preterm (64%) and low-birth-weight infants (68%). While the results from blood culture and PCR were comparable in EOS cases, PCR demonstrated significantly higher bacterial detection rates compared to blood culture (85.3% vs. 68%, respectively, *p* = 0.001). Blood culture exhibited 100% specificity, whereas 16S rRNA PCR exhibited greater sensitivity, enabling rapid and accurate diagnosis. For antimicrobial sensitivity testing, blood culture remained the most precise method [34].

In research conducted by Hayder Hamad et al., PCR was assessed as a diagnostic method for identifying neonatal sepsis, comparing its efficacy to blood culture in 85 neonates suspected of sepsis. The molecular detection of bacterial sepsis involved using specific primers for 16S rRNA and rpoB genes. Notably, 20% of the samples exhibited the presence of 16S rRNA genes, while the rpoB gene was present in 18.8%. Interestingly, among the neonates with a positive PCR result for 16S rRNA, 11 had negative bacterial blood culture results [35]. The rpoB gene primers were utilized to identify Enterobacteriaceae in neonatal blood samples [35]. It is important to note that the studies analyzed in this meta-analysis did not differentiate between EOS and LOS. Additionally, the challenge of lacking a universally accepted definition of sepsis is a limitation of this study too.

In a recent meta-analysis encompassing 19 studies, the evaluation of 16S rRNA PCR testing for EOS demonstrated a sensitivity of 0.98, specificity of 0.94, positive likelihood ratio of 16.0, negative likelihood ratio of 0.02, diagnostic odds ratio of 674, and an AUC of 0.99. These results underscore the effectiveness of 16S rRNA PCR testing for the prompt diagnosis of neonatal sepsis [36]. The advantages of 16S rRNA PCR over blood culture include its ability to detect bacteria even at low bacterial loads, independence from previous antibiotic exposure, and the capability to identify species challenging to culture in blood culture [37]. However, it has a high cost and, despite methods developed to mitigate contamination [38,39,40], it is susceptible to contamination from clinical collection, laboratory reagents, or the laboratory environment, potentially dominating the sequence results, especially in neonates with low levels of bacteremia [21,28].

While the 16S rRNA gene shows promise for bacterial identification, its use is limited for closely related species due to insufficient sequence diversity [41]. In contrast, the region between the 16S and 23S rRNA gene loci, being under less evolutionary pressure, exhibits greater genetic variation [42], enabling the discrimination of closely related strains [43]. Recent studies often prefer the use of the region between the 16S and 23S rRNA gene loci in PCR techniques, as it offers a more rapid and cost-effective sequencing alternative. This technique, mentioned by various names such as molecular multiplex PCR or molecular culture, was discussed in the studies reviewed here.

### 3.3. Molecular Multiplex PCR

A recent investigation involving 229 neonates, encompassing 40 very-low-birth-weight and 159 preterm infants, evaluated the molecular multiplex PCR test (ROCHE Light Cycler SeptiFast^®^ (Roche Diagnostics, Penzberg, Germany)—a test no longer commercially available). This test, which targeted sequences between bacterial 16S–23S ribosomal RNA and fungal 18S-5.6S ribosomal RNA, could identify 20 common microbial agents associated with 90% of blood infections in both adults and pediatric cases [20]. The study revealed a higher sensitivity of the PCR test compared to blood culture, with similar specificity as well as negative and positive predictive values. However, the PCR test generated more false positives, likely attributed to sampling contamination, particularly involving pathogens unrelated to EOS. Despite its rapid result availability, the study did not recommend the use of PCR for EOS diagnosis, a conclusion that agreed with the conclusions drawn from an earlier systematic review [28]. Notably, the molecular PCR failed to enhance diagnostic accuracy, especially in low-risk infants who undergo infection screening [20].

### 3.4. Molecular Culture

In a more recent study, involving a notably smaller cohort of 38 neonates, an unrestricted PCR-based technique was assessed. This method detects and identifies bacterial DNA through the 16S–23S rRNA gene interspace regions, termed as rapid molecular culture [44]. Interestingly, blood culture yielded negative results for all participants, while molecular culture indicated positivity in three cases (7.5%). One of these cases was identified as clinical EOS, while the remaining two were considered false positives. The advantages of molecular culture included its capability to detect bacteria, even with a low bacterial load, with results available within 4 h. Moreover, molecular culture successfully identified Enterococcus faecalis in a case of clinical EOS with negative blood culture, showcasing its effectiveness in detecting microorganism challenges to identify using conventional techniques. The authors suggested that molecular culture could potentially replace traditional blood culture in EOS diagnostics, providing guidance to clinicians on the appropriate timing to discontinue antibiotic therapy shortly after birth [44]. However, it is crucial to acknowledge several limitations of this study. Firstly, the sample size was limited, and there were no cases of culture-positive EOS, leaving uncertainty regarding the ability of molecular culture to predict a positive blood culture. Additionally, despite clinician training for sterile sample collection, the possibility of contamination during collection or analysis remained a concern.

### 3.5. Sequencing the Bulk DNA and/or RNA

An alternative approach involves sequencing the entire DNA and/or RNA content in a given sample. Following the exclusion of human DNA or RNA, the remaining sequences can be scrutinized to identify bacteria, viruses, fungi, and parasites [21,45]. The extensive sequencing of total DNA and RNA holds particular appeal for its capability to impartially detect all potential pathogens, including the detection of polymicrobial infections. Nevertheless, this approach has various challenges, encompassing the potential introduction of biases during sample preparation, limitations in sequencing depth, and a lack of standardized software tools and pipelines for sequence analysis [46,47]. Additionally, drawbacks include concerns related to contamination and the absence of suitable negative controls. It is important to note that these innovative techniques are linked to elevated costs and often require sophisticated technology and specialized laboratory facilities [21].

In conclusion, various novel PCR techniques aim at identifying microorganisms. The available studies compared these techniques with the gold standard, blood culture. From the current results, it seems that these novel techniques, although having many advantages, cannot replace blood culture.

## 4. Whole-Genome Sequencing

Whole-genome sequencing is not a technique ready to be implemented in routine diagnostics due to the high cost, technical optimization required, and bioinformatic and statistical expertise needed to analyze complex sequencing data [21].

## 5. MicroRNAs in EOS

miRNAs are short RNA molecules, approximately 22 nucleotides in length, lacking protein-coding capability but regulating gene expression by inhibiting the translation or transcription of target mRNAs. They act through binding to the 3 UTR region of the target mRNA [48]. To date, over 2000 miRNAs have been identified in the human genome [49]. Post-transcriptional regulation involving various miRNAs plays a role in the pathophysiology of sepsis [50]. Consequently, numerous studies have explored their potential as diagnostic biomarkers in EOS [51,52,53,54,55,56,57]. Although not able to detect microorganisms directly, they have been studied for their diagnostic accuracy and potential utility as diagnostic markers for EOS. miRNAs offer advantages such as ease of analysis in small blood volumes and remarkable stability in biospecimens [58,59].

Several miRNAs have been investigated as diagnostic markers in neonatal sepsis, encompassing both EOS and LOS. A recent systematic review and meta-analysis indicated the beneficial role of miRNA detection in neonatal sepsis diagnosis, with a pooled specificity of 0.76 and pooled sensitivity of 0.83, demonstrating relatively high overall accuracy [60]. In this study, the pooled diagnostic odds ratio was 15.81, which is close to that of traditional markers. This discussion focuses on miRNAs studied specifically in EOS, and the information is summarized in Table 2.

miR-15a and miR-16 emerged as crucial diagnostic biomarkers for neonatal sepsis [62]. Zhao et al. found moderate diagnostic value for miR-15a-5p and miR-16-5p in EOS [56], suggesting their involvement in the inflammatory process by activating the NF-κB pathway and targeting TNIP2 [63]. A Study by Fouda et al. highlighted the significance of miR-15b and miR-378a, demonstrating their potential as discriminators of sepsis, with high sensitivity and specificity [55].

A recent study showed the diagnostic potential of miR-16a in neonates with sepsis (ages 1–11 days) [52]. This study included both neonates with EOS and LOS and found that neonates with sepsis had considerably higher levels of miRNA-16a and miRNA-451 than the healthy neonates (*p* ≤ 0.001). miR-16a exhibited superiority to miRNA-451 in terms of sensitivity and specificity [52].

miR-26a, implicated in immune response regulation, displayed decreased expression in neonates with sepsis. An association was also found between the downregulation of miR-26a and the overexpression of IL-6 in blood mononuclear cells [64]. The study of Zhao and Zhang in 51 septic neonates agreed that miR-26a had lower expression compared to healthy newborns. Therefore, lower baseline miR-26a expression indicated the occurrence of EOS [57]. Furthermore, this study found that the expression of miR-26a in newborns with EOS was significantly higher 72 h after birth than within 72 h of birth. This information indicates that miR-26a might be involved in the progression of EOS or in some protective mechanisms against EOS. Both the above studies evaluating miR-26a had a small number of neonates.

miR-223 and miR-132, involved in negative regulation of the inflammatory response, were downregulated in EOS, possibly linked to an increased expression of immune-related genes in the Toll-like receptor (TLR) signaling pathway [51]. Furthermore, a retrospective case–control study identified miR-211-5p and miR-223-5p as overexpressed miRNAs in EOS, demonstrating good discrimination potential [54].

In the study of El-Khazragy et al., of 70 neonates with EOS, miR-1 and miR-124 were increased and were significantly associated with sepsis, while miR-34a expression was reduced. miR-34a exhibited the highest specificity (97%) as a confirmatory test for neonatal sepsis. In the multivariate model, miR-1 and miR-124 were found to be significant predictors of disease progression or mortality [53].

miR-23b levels were found to be crucial in EOS development, exhibiting a negative correlation with mortality, since levels have been shown to be markedly downregulated in blood cultures of newborns who died from EOS. This suggested that this miRNA could serve both as a diagnostic marker and as a tool to monitor sepsis progression [61].

Combining specific miRNAs, such as miR-223-3p, miR-15a-5p, and miR-16-5p, demonstrated enhanced diagnostic value for EOS [56].

Despite the insights gained from various studies, many studies are single-center, and the total number of patients included was relatively small in each study. There is a need for larger, multicenter studies with a higher number of patients to validate the diagnostic efficacy of miRNAs in neonatal sepsis accurately.

## 6. T2 Magnetic Resonance (T2MR) Technology

T2MR is a diagnostic technology designed for the rapid detection of pathogens directly from whole blood samples, without the need for blood culture. It is a direct molecular assay that utilizes magnetic resonance technology to identify the presence of pathogens in the bloodstream.

The study of Lucignano evaluated both T2Bacteria and T2Candida panels in a cohort of 754 children, which included 27 neonates [65]. These nanodiagnostic panels have the capability to detect clinically relevant pathogens directly from a single fresh whole blood specimen, eliminating the need for prior blood culture. The entire process is fully automated, requiring only a few hours of laboratory turnaround time. The T2MR technology’s efficient assay workflow allows for the specific identification of circulating live pathogens, whether free or encapsulated within white blood cells. This feature helps prevent false-positive results associated with freely circulating DNA.

The sensitivity and specificity of the T2Bacteria and T2Candida panels were reported as 84.2% and 100%, with 85.9% and 94.1%, respectively. Notably, the sensitivity and specificity of the T2Bacteria panel increased to 94.9% and 98.7%, respectively, when blood culture yielded negative results, but other laboratory data supported the molecular findings [65]. In this cohort study, both panels demonstrated a significant advantage over blood culture in terms of time to identification, while ensuring consistent sensitivity and specificity. It is essential to highlight that this study, albeit satisfactory in its inclusion of children and neonates, is retrospective in nature. Therefore, these promising results warrant validation through larger-scale studies for conclusive confirmation.

## 7. Bioinformatic Analysis

Bioinformatic analysis plays a crucial role in unraveling the underlying mechanisms of neonatal sepsis by examining datasets related to gene expression. During the initial phases of neonatal sepsis, a cascade of events occurs, involving immune cells like monocytes and macrophages, along with the release of inflammatory mediators and cytokines. This complex interplay leads to an exaggerated inflammatory response. Conversely, as neonatal sepsis progresses to its later stages, there is a notable shift toward immunosuppression becoming the prevailing characteristic [66].

Through the bioinformatic analysis of published transcriptional data, NSUN7, PROS1, TDRD9, RETN, LOC728401, and METTL7B were identified as potential biomarkers of neonatal sepsis from the perspective of immune cell infiltration combined with logistic regression. More importantly, the developed diagnostic models provide a new perspective for future research on the pathogenesis of neonatal sepsis [67].

The study of Bai et al. also aimed at discovering reliable biomarkers for the diagnosis of neonatal EOS through transcriptomic analysis of publicly available datasets. Four genes, CST7, CD3G, CD247, and ANKRD22, were identified that most accurately predicted neonatal EOS and were subsequently used to construct a diagnostic model. ROC analysis revealed that this diagnostic model performed well in differentiating between neonatal EOS and normal infants [68]. In addition, when compared with conventional inflammatory indicators, such as CRP, hemoglobin, neutrophil count, and PCT, the model had better diagnostic performance.

## 8. Conclusions

PCR-based techniques, especially unrestricted methods utilizing 16S rRNA sequencing and 16S–23S rRNA gene interspace region sequencing, offer notable advantages. They can detect bacteria, even at low loads, identify species not easily cultured, and provide rapid results. Despite these advantages, these techniques present challenges, such as susceptibility to sample contamination, high costs, and the need for specialized laboratories and personnel. Several miRNAs may also have significant diagnostic utility in EOS. Molecular testing, when integrated into the diagnostic algorithm for EOS, particularly in conjunction with clinical judgment and routine laboratory parameters, could prove valuable. The currently available molecular assays may not have sufficient diagnostic accuracy to replace microbial cultures. They can be used as adjuncts to overcome some difficulties in blood culture and to help in clinical decisions.

Innovative techniques like T2 magnetic resonance and bioinformatic analysis of published data are areas of ongoing investigation for their potential application in neonatal sepsis. While none of these approaches has proven sufficient to replace blood culture, they can be effectively employed as adjuncts to enhance the diagnostic process.

## Figures and Tables

**Table 1:** Advantages and disadvantages of novel PCR techniques used for identifying microorganisms.

Advantages	Disadvantages
1. Detection of small quantity of microbial (bacterial or fungal) DNA or RNA 2. Rapid results within hours 3. Identification of a variety of pathogens (detection of polymicrobial infections) 4. Identification of pathogens not easily cultured	1. False-positive results due to contamination 2. Not available information on antibiotic susceptibility 3. High cost 4. Certain techniques require specialized laboratories and personnel.

**Table 2:** miRNAs that have been evaluated in early-onset sepsis.

miRNA	Number of Patients vs. Controls	AUC	Sensitivity	Specificity	Levels	Study
miR-15a-5p	43 vs. 59	0.67			Downregulated	Zhao et al., 2023 [56]
miR-15b	25 vs. 25		76%	88%	Upregulated	Fouda et al., 2021 [55]
miR-16-5p	43 vs. 59	0.68			Upregulated	Zhao et al., 2023 [56]
miR-16a	25 vs. 25	0.968	88%	98%	Upregulated	El-Hefnawy et al., 2021 [52]
miR-451	25 vs. 25		64%	61%	Upregulated	El-Hefnawy et al., 2021 [52]
miR-378a	25 vs. 25		60%	88%	Downregulated	Fouda et al., 2021 [55]
miR-26a	51 vs. 102				Downregulated	Zhao and Zhang, 2021 [57]
miR-223	25 vs. 25				Downregulated	Dhas et al., 2018 [51]
miR-223-5p	20 vs. 21	0.988			Upregulated	Ernst et al., 2021 [54]
miR-132	25 vs. 25				Downregulated	Dhas et al., 2018 [51]
miR-211-5p	20 vs. 21	0.787			Upregulated	Ernst et al., 2021 [54]
miR-34a	70 vs. 70	0.94	89%	97%	Downregulated	El-Khazragy et al., 2023 [53]
miR-1	70 vs. 70	0.82	81%	83%	Upregulated	El-Khazragy et al., 2023 [53]
miR-124	70 vs. 70	0.85	86%	83%	Upregulated	El-Khazragy et al., 2023 [53]
miR-23b	27 vs. 13				Downregulated in neonates who died of sepsis Upregulated in neonates who survived	Fatmi et al., 2020 [61]
Combination of miR-15a-5p, miR-223-3p, and miR-16-5p	43 vs. 59	0.85	74.6%	86%	miR-15a-5p and miR-233-3p downregulated miR-16-6p upregulated	Zhao et al., 2023 [56]
